# Distinct distribution patterns of ammonia-oxidizing archaea and bacteria in sediment and water column of the Yellow River estuary

**DOI:** 10.1038/s41598-018-20044-6

**Published:** 2018-01-25

**Authors:** Mingcong Li, Guangshan Wei, Wenchong Shi, Zhongtao Sun, Han Li, Xiaoyun Wang, Zheng Gao

**Affiliations:** 1 State Key Laboratory of Crop Biology, Shandong Agricultural University, Tai’an, 271018 China; 2 College of Life Sciences, Shandong Agricultural University, Tai’an, 271018 China; 3 Department of Botany and Microbiology, Institute for Environmental Genomics, University of Oklahoma, Norman, OK USA; 4 Key Laboratory of Marine Genetic Resources, Third Institute of Oceanography, SOA, Xiamen, 361005 China; 5 South China Sea Resource Exploitation and Protection Collaborative Innovation Center (SCS-REPIC), Sun Yat-Sen University, Guangzhou, 510275 China

## Abstract

Ammonia oxidation is a critical process of estuarine nitrogen cycling involving ammonia-oxidizing archaea (AOA) and bacteria (AOB). However, the distribution patterns of ammonia-oxidizing microorganisms (AOMs) between different habitats in the same area remain unclear. The present study investigated the AOMs’ abundance and community compositions in both sediment and water habitats of the Yellow River estuary. Quantitative PCR (qPCR) revealed that AOA showed significant higher abundance than AOB both in sediment and water samples. AOA and AOB abundance distribution trends were consistent in sediment but distinct in water along the sampling sites. Clone library-based analyses showed that AOA sequences were affiliated with *Nitrososphaera, Nitrosopumilus* and *Nitrosotalea* clusters. Generally, *Nitrososphaera* was predominant in sediment, while *Nitrosopumilus* and *Nitrosotalea* dominated in water column. AOB sequences were classified into genera *Nitrosospira* and *Nitrosomonas*, and *Nitrosospira* dominated in both habitats. Principal coordinate analysis (PCoA) also indicated AOA community structures exhibited significant differences between two habitats, while AOB were not. Ammonium and carbon contents were the potential key factors to influence AOMs’ abundance and compositions in sediment, while no measured variables were determined to have major influences on communities in water habitat. These findings increase the understanding of the AOMs’ distribution patterns in estuarine ecosystems.

## Introduction

Ammonia oxidation, the first and rate limiting step of nitrification process, which can remove a substantial percentage (10–80%) of anthropogenic nitrogen pollution and reduce the risk of eutrophication in estuaries when coupled with denitrification^1–3^. Ammonia-oxidizing bacteria (AOB) were thought to be largely responsible for the oxidation of ammonia to nitrite in the past time^4^. The known AOB are categorized into two distinct monophyletic, *Betaproteobacteria* (β-AOB) from the genera *Nitrosomonas* and *Nitrosospira*, and *Gammaproteobacteria* (γ-AOB) from the genus *Nitrosococcus*^5–8^. However, this long-held view has been changed since the first cultivated ammonia-oxidizing archaea (AOA), *Nitrosopumilus maritimus*, which was isolated from a marine aquarium tank^9^. The identification of ammonia monooxygenase (AMO) in *Thaumarchaeota* indicated that both the AOA and AOB can responsible for the conversion of ammonia to hydroxylamine^10,11^. From then on, large number of researches investigated on the abundance, diversity and community structure of AOA and AOB in various environments, indicating their widely distributions in both marine and terrestrial ecosystems^12,13^.

The community structure and spatial variation of AOA and AOB are essential for assessing the nitrification process. It has been suggested that the distribution of AOA and AOB diversity could be dependent on the types of habitat^12^. Previous studies indicated that AOA showed more abundance than AOB in many ecosystems such as paddy soils^14^, acidic soils^15^, river sediment and freshwater Lakes^16,17^. Whereas bacteria rather than archaea are more active ammonia oxidizers in N-rich grassland soil^18^, Qinghai Lake^19^ and Yangtze Estuary^20^. These differences may be due to the distinct habitat types and the effect of environmental factors.

The abundance and diversity of AOA and AOB can also be influenced by the variation of environmental factors. Many studies demonstrated a wide range of abiotic factors shaping the AOM distribution patterns, such as pH^14,21^, salinity^22,23^, ammonium (NH_4_^+^-N) and organic matter^16,24,25^, as well as spatial and temporal factors^26^. As the substrate of ammonia oxidation, ammonia has been considered as a primary factor to manipulate AOMs’ distribution. A previous study reported that the growth of AOB was favored at a high level of ammonium in agricultural soils^24^. Two followed studies about freshwater rivers demonstrated that the community distribution of ammonia-oxidizers showed to be clearly related to NH_4_^+^-N and organic matter^16,25^. pH is another important factor to influenced the distribution of AOMs^14,27^. It is reported that AOA are increasingly recognized as the primary mediators of ammonia oxidation in acidic soils^27^. In aquatic environments, salinity is another important factor can affect the structure and abundance of ammonia oxidizing microbial community, particularly in estuary ecosystem^22,23^. Although niches separation and environmental effects are two critical factors that shape ammonia-oxidizing microbial communities, their relative influence remains controversial.

Estuary is determined as an ideal habitat for AOMs by dozens of researches, a number of studies chose estuary as research media to reveal the key factors of influencing the distributions and possible functions of ammonia-oxidizing microorganisms^3,20,28,29^. However, most published surveys have only conducted on estuary sediment or water ecosystem, and the different distribution patterns of AOA and AOB between sediment and water column remain unclear.

The Yellow River estuary located at the interface of the Yellow River and the Bohai Sea, is the largest turbid river in the world^30^. It carries a large amount of sediments, pollutions and nutrients from the river and coastal land every year. Our previous study has demonstrated that the dominant bacteria and archaea of the Yellow River Estuary were mostly related to carbon, nitrogen, and sulfur cycling processes, such as methanogenesis, ammonia oxidation and sulfate reduction^31^. Whereas the microorganisms responsible for nitrification in this area remain unknown. In this study, surface sediments and their overlying water samples were collected from different stations around the estuary. The objectives of this study were (i) to elucidate the community structure and abundance of AOA and AOB in the estuary environment, (ii) to determine whether AOM show different assembly patterns in two habitats and (iii) to identify the key environmental factors influencing the niche segregation for AOA and AOB in Yellow River Estuary. This study will provide insights for our understanding and knowledge about the ecology of AOMs in different habitats of temperate estuarine ecosystems.

## Result

### Abundance of AOA and AOB *amoA* genes

**Figure 1:**
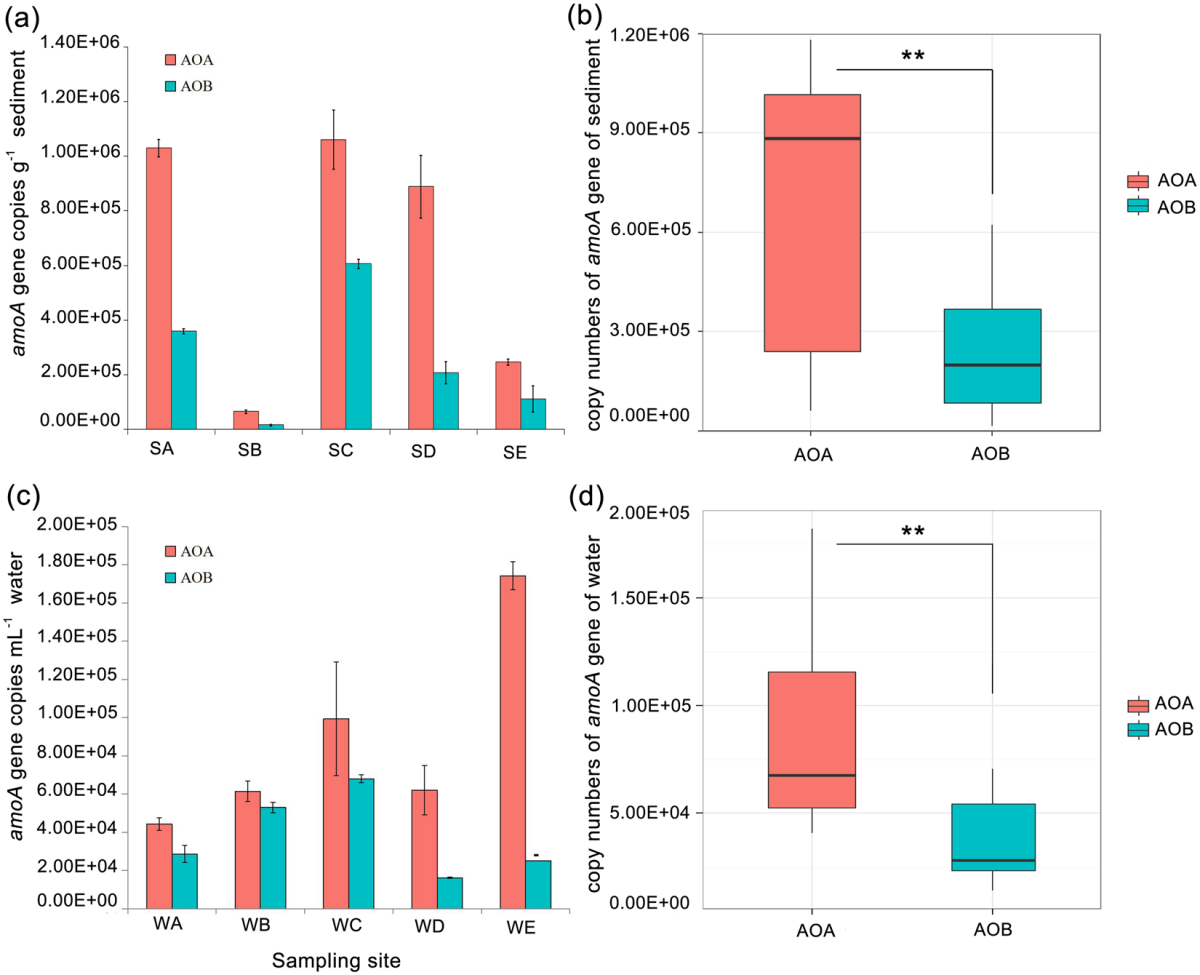
Copy numbers of AOA and AOB *amoA* genes in sediment and water samples of the Yellow River estuary. (**a**) The data are presented as the mean ± SE of three independent experiments in each sediment site. (**b**) Box plots represent the differences in abundance between AOA and AOB *amoA* gene copies in sediment. (**c**) The data are presented as the mean ± SE of three independent experiments in each water site. (**d**) Box plots represent the differences in abundance between AOA and AOB *amoA* gene copies in water.

The abundance of AOA and AOB *amoA* genes estimated by quantitative PCR is shown in Fig. 1. In sediment, the abundance of AOA and AOB *amoA* genes ranged from 6.53 ± 0.67 × 10^4^ to 1.06 ± 0.11 × 10^6^ and 1.61 ± 0.28 × 10^4^ to 6.07 ± 0.18 × 10^5^ copies per gram wet sediment among sites, respectively (Fig. 1a). In water, AOA and AOB *amoA* gene copy numbers ranged from 4.43 ± 0.33 × 10^4^ to 1.74 ± 0.07 × 10^5^ and 1.61 ± 0.21 × 10^4^ to 6.80 ± 0.27 × 10^4^ copies per ml water, respectively (Fig. 1c). The AOMs copies showed relative higher abundance in sediment than that of water column. Both in sediment and water habitats, the AOA *amoA* genes performed significantly higher abundance than that of AOB (*P* < 0.01, Fig. 1b and d). In comparison among the sampling sites, archaeal and bacterial ammonia oxidizers abundance showed extreme similar distribution patterns (Spearman correlation analysis, *P* < 0.001) in sediment, with relative higher abundance in sites SC, SA and SD than that of sites SB and SE (Fig. 1a). While on the contrary, AOA and AOB *amoA* gene abundance showed distinct distribution trends along sampling sites in water column (Spearman correlation analysis, *P* > 0.05, Fig. 1c).

### Diversity of AOA and AOB communities

The α- and β-diversity of ammonia-oxidizing communities were investigated using the method of *amoA* gene clone library (Table 1). Based on a 85% similarity cutoff reported in previous studies^16,32^, a total of 457 archaeal *amoA* gene clone sequences were grouped into 14 OTUs, and 506 bacterial *amoA* gene sequences were assigned into 20 OTUs. Both in sediment and water habitats, AOB showed higher α-diversity (Chao1, Shannon indexes) in seawater sites SA, SC and SD than that of AOA. While in freshwater site WE, AOA α-diversity were higher than AOB. In the comparison between sediment and water at the same sampling sites, AOA performed higher Shannon index in sediment samples than that of water samples. Conversely, AOB showed higher diversity in most water samples than that of sediment.

**Table 1:** Descriptions of clone libraries and diversity indices of the archaeal and bacterial *amoA* gene sequences from each sampling site.

Sampling site	*amoA* sequence/No. of OTUs		Chao1 index		Shannon index		Coverage (%)	
	AOA	AOB	AOA	AOB	AOA	AOB	AOA	AOB
SA	73/7	65/9	7.0	10.0	1.39	1.55	100.00	95.38
SB	49/6	40/3	6.0	3.0	1.26	0.44	100.00	97.50
SC	79/9	39/9	12.0	14.0	1.57	1.48	96.20	87.18
SD	42/4	50/10	5.0	10.3	0.53	1.91	95.24	96.00
SE	43/8	80/6	8.0	6.0	1.87	1.23	97.67	98.75
WA	32/7	53/9	10.0	11.0	1.17	1.58	87.50	92.45
WB	33/6	46/12	6.5	13.2	1.13	2.06	93.94	91.30
WC	45/7	38/13	8.5	41.0	1.06	2.13	93.33	78.95
WD	34/3	57/10	3.0	13.0	0.43	1.57	97.06	92.98
WE	27/8	38/9	14.0	11.0	1.75	1.70	85.19	89.47

**Figure 2:**
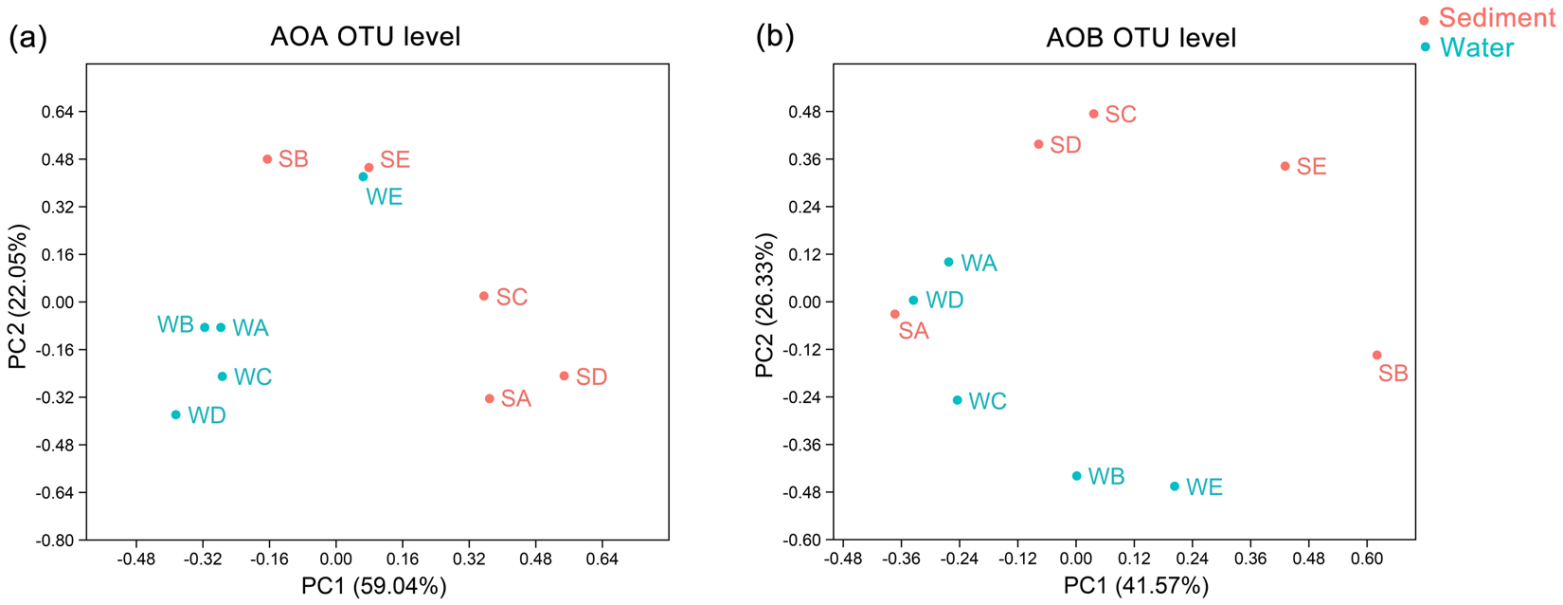
PCoA plots at OTU level of (**a**) AOA and (**b**) AOB in sediment and water samples.

Principal coordinate analysis (PCoA) and analysis of similarities (ANOSIM) tests were used to evaluate β-diversity and community similarity of ammonia-oxidizers in Yellow River estuary (Fig. 2). The OTU-based PCoA results indicated that the AOA community exhibited significant difference between sediment and water (R = 0.61, *P* = 0.009, ANOSIM test), but AOB community showed no significant difference (R = 0.22, *P* = 0.112) between habitats. The water AOA community at site E (WE) showed greater similarity with the sediment sites (SE and SB), indicating that water from the Yellow River had an obvious influence on the sediment microbial communities near the estuary.

### Phylogenetic analysis and community structure of AOA

**Figure 3:**
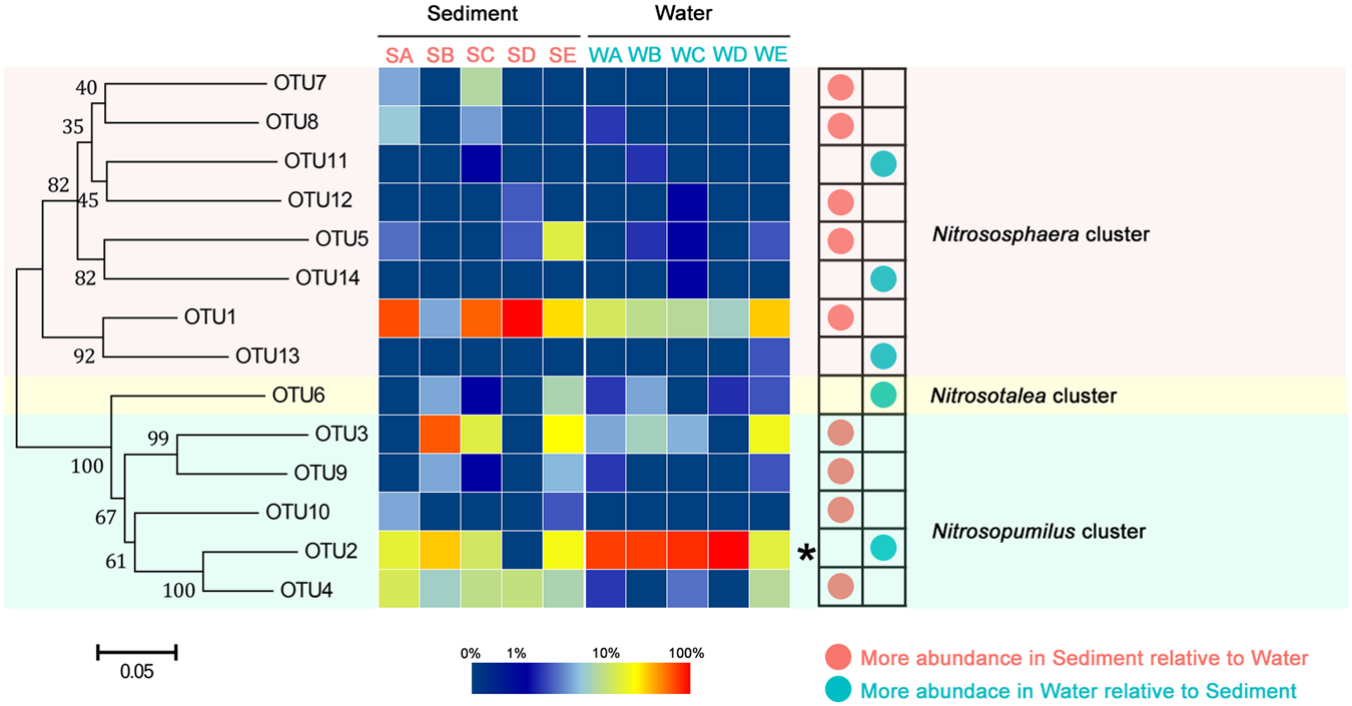
Neighbor-joining phylogenetic tree and community distributions of AOA *amoA* gene sequences from the Yellow River estuary. Bootstrap values greater than 50% of 1,000 replicates are shown, and the scale bar represents 5% sequences divergence. Values of each OTU relative proportion are color coded in the corresponding heat map legends.

The phylogenetic tree of AOA was constructed with *amoA* gene sequences of representative OTUs and known AOA isolated strains (Fig. 3 and Fig. S1). The phylogenetic tree indicated that all retrieved sequences could be categorized into three clusters, the *Nitrososphaera*, *Nitrosopumilus* and *Nitrosotalea*. The *Nitrosopumilus* cluster, with 5 OTUs and 255 clones (55.80% of the total AOA sequences), was the most abundant branch of AOA in the Yellow River estuary; followed by the *Nitrososphaera* cluster, with 8 OTUs and 191 clones (41.79%), whereas the *Nitrosotalea* cluster was very rare, only with 1 OTUs and 11 clones (2.41%). The *Nitrososphaera* and *Nitrosopumilus* clusters were detected in all sampling sites, indicating their widespread distributions in this estuary. Whereas *Nitrosotalea* showed more geographical limitation, can be found in all sites except SA, SD and WC.

Cluster and community composition analyses were performed to demonstrate the community structures of ammonia-oxidizing microorganisms in the Yellow River estuary (Fig. S3). The Cluster analysis showed that AOA community can be clustered into two separate branches (Fig. S3a). Sediment samples belonging to seawater sites (SA, SC and SD) were grouped together, and other samples formed another branch. The *Nitrososphaera* cluster was the absolutely dominant AOA in sediment samples SA, SC and SD, which accounted for 68.49%, 63.29% and 90.48%, respectively. Whereas for other samples, the *Nitrosopumilus* cluster occupied more than a half percentage in each site.

### Phylogenetic analysis and community structure of AOB

**Figure 4:**
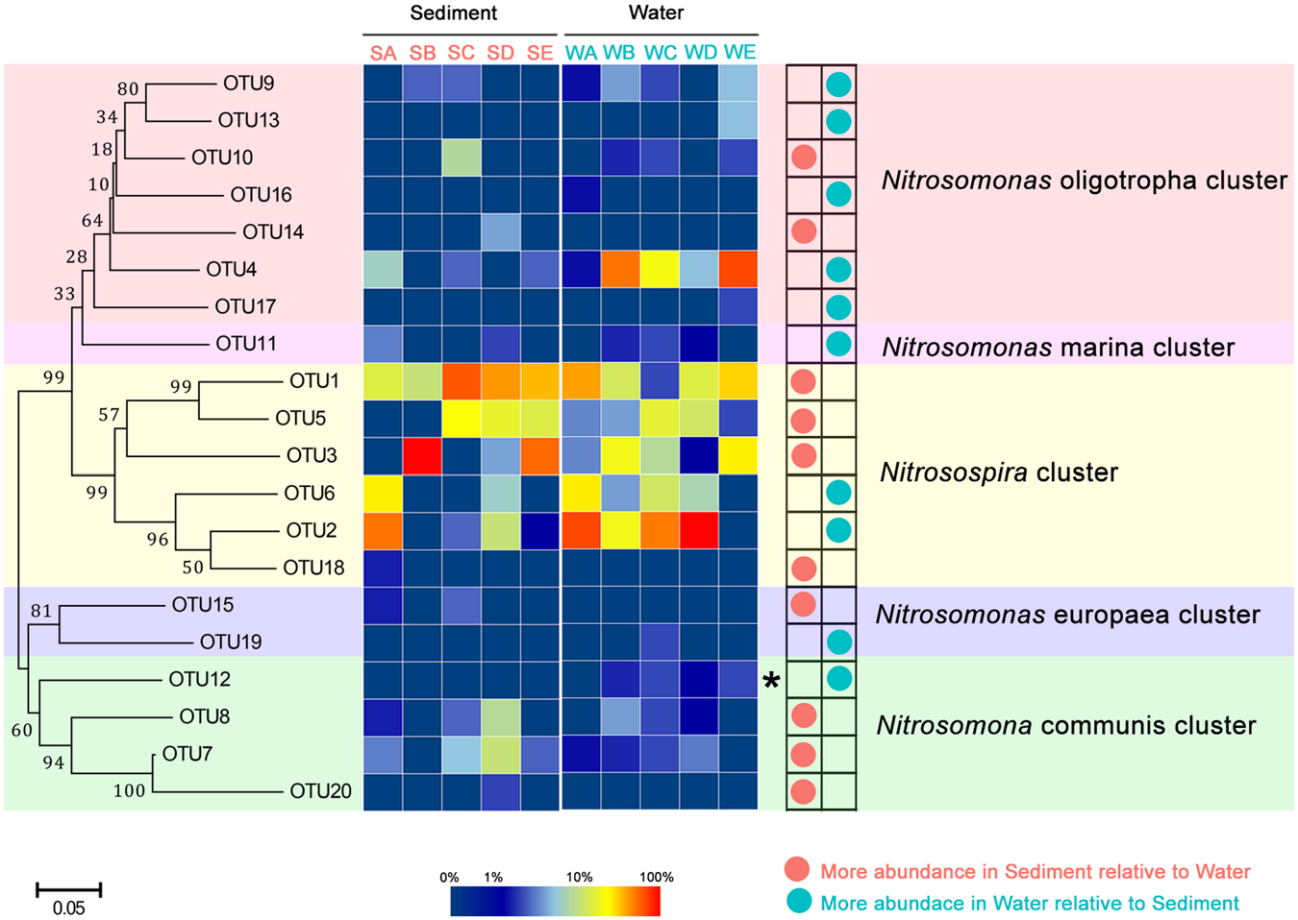
Neighbor-joining phylogenetic tree and community distributions of AOB *amoA* gene sequences from the Yellow River estuary. Bootstrap values greater than 50% of 1,000 replicates are shown, and the scale bar represents 5% sequence divergence. Values of each OTU relative proportion are color coded in the corresponding heat map legends.

The phylogenetic tree of the AOB *amoA* genes showed that all retrieved sequences were categorized into two major genera, *Nitrosomonas* and *Nitrosospira* (Fig. 4 and Fig. S2). The genus *Nitrosomonas* included four clusters: *Nitrosomonas marina*, *Nitrosomonas oligotropha*, *Nitrosomonas europaea* and *Nitrosomonas communis*. The genus *Nitrosospira* was the most abundant group, which accounted for 78.85% of all 506 bacterial *amoA* gene sequences with 6 OTUs, and followed by the *N. oligotropha*, with 7 OTUs and 67 clones (13.24%). The *N. europaea* was the rarest group, with 2 OTUs and 3 clones (0.59%). The *Nitrosospira* and *N. oligotropha* were found in all sampling sites, indicating their widespread distributions in both water and sediment habitats. Whereas *N. europaea* cluster showed more geographical limitation, which were only detected in samples SA, SC and WC.

The similarity analysis showed that AOB community can be clustered into two separate branches, water samples WB and WE formed in one and others formed another (Fig. S3b). The genus *Nitrosospira* as the most abundant group, accounts for more than half percentage in all sites (65.79% to 92.45%) except water samples WB and WE (50.00% and 42.11%). Furthermore, *N. oligotropha* cluster showed relative more ratios (39.13% and 55.26%) in water samples WB and WE contrast with other samples.

### Relationships between AOM communities and environmental factors

**Figure 5:**
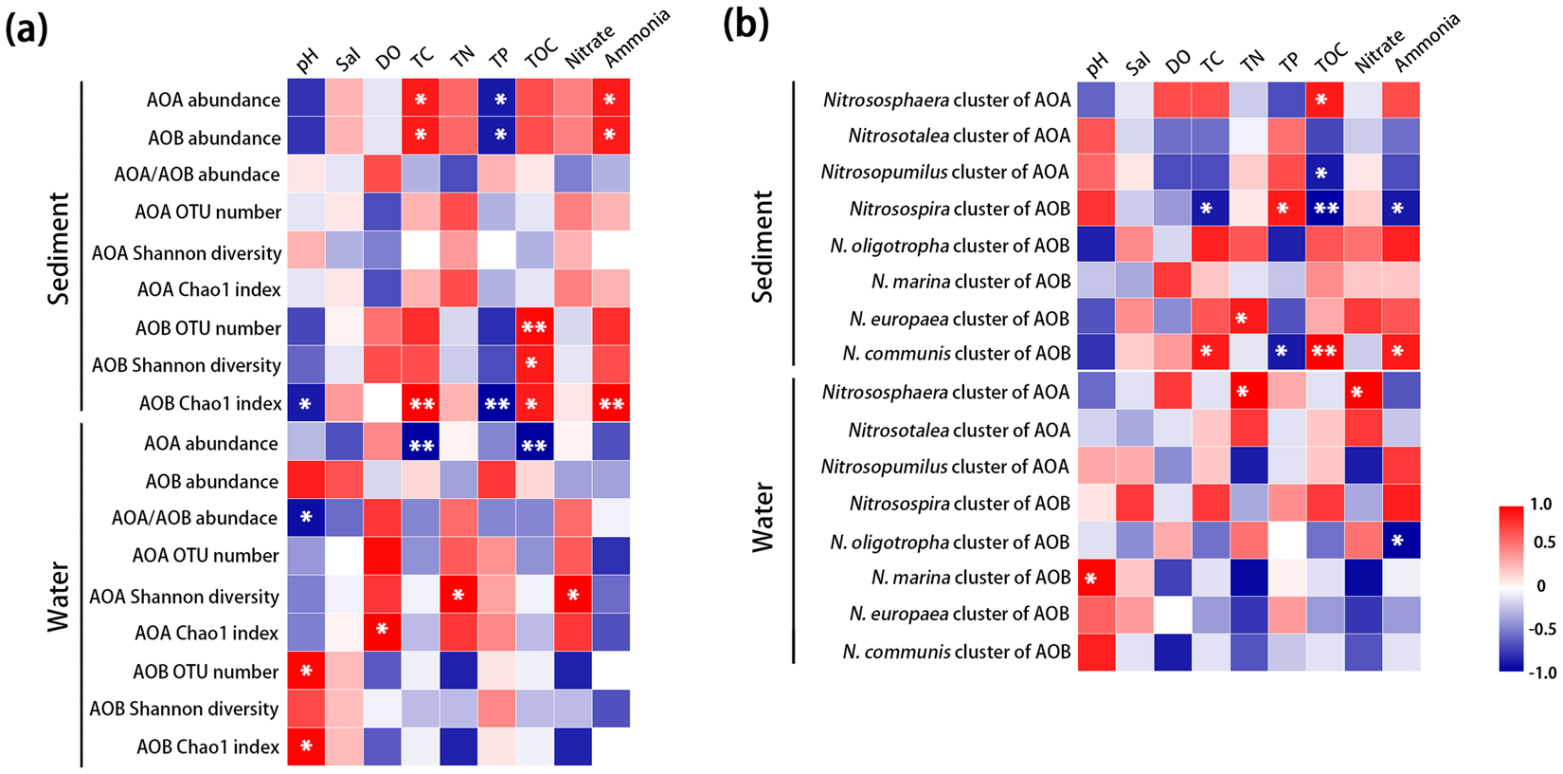
Heat maps demonstrate correlations between ammonia-oxidizing microbial abundance, α-diversity, dominant taxa (cluster and genus) and environmental factors. Values of correlation coefficients are color coded in the corresponding heat map legends. Correlations are significant at the 0.05 level (*) and the 0.01 level (**) using a two-tailed test.

Spearman correlation analyses were performed to reveal the relationships between ammonia-oxidizing microbial abundance, α-diversity, dominant taxa and environmental factors (Fig. 5). In sediment, both archaeal and bacterial *amoA* gene abundances were significantly and positively correlated with the concentrations of total carbon (TC, *P* < 0.05) and ammonium (*P* < 0.05), but they were negatively correlated with the concentrations of total phosphorus (TP, *P* < 0.05, Fig. 5a). The AOB α-diversity, including OTU number, Shannon diversity and Chao1 index, were significantly and positively correlated with the total organic carbon concentrations (TOC, *P* < 0.05). In addition, AOB Chao1 index was also notably and positively correlated with concentrations of TC and ammonium (*P* < 0.01), but has significantly negative correlations with the TP (*P* < 0.01) and pH (*P* < 0.05). Intriguingly, no significant correlations were detected between AOA α-diversity and measured environmental factors indicating the different responses of AOA and AOB to environmental influences.

In water, the AOA abundance had significantly negative correlations with concentrations of TC and TOC (*P* < 0.01). Additionally, the ratio of AOA/AOB *amoA* gene abundance existed a negative correlation with pH (*P* < 0.05). The AOA Shannon index was notably and positively correlated with total nitrogen (TN) and nitrate concentration (*P* < 0.05), and Chao1 index showed positive correlation with TC (*P* < 0.05). Both the OTU number and Chao1 index of AOB were significantly and positively correlated with pH value (*P* < 0.05, Fig. 5a).

Relationships between AOM dominant taxa and environmental factors were shown in Fig. 5b. In sediment, the *Nitrososphaera* cluster (AOA) was significantly and positively correlated with the concentration of TOC (*P* < 0.05), whereas the *Nitrosopumilus* cluster (AOA) performed negative relationships (*P* < 0.05). The *Nitrosospira* (AOB) genus was significantly and negatively correlated with TC (*P* < 0.05), TOC (*P* < 0.01) and ammonium concentrations (*P* < 0.05), whereas *Nitrosomonas* genus (include *N.oligotropha, N.marina, N.europaea* and *N.communis* of AOB) showed positive relationships with these factors. Only few significant correlations were detected between AOM species and environmental factors in water column (Fig. 5b).

Mantel test was used to evaluate the relationship between AOMs community compositions (cluster level) and environmental factors (Table S3 and S4). According to Mantel test, TC and TOC contents have significant correlations with the sediment AOA and AOB community compositions (*P* < 0.05). However, there was no notable correlation between environmental factors and water AOMs compositions. Similarly, redundancy analysis (RDA) based on AOMs community structures and environmental factors also confirmed that carbon significantly influenced the sediment AOMs’ distribution (Fig. S4a and S4b). Concretely, TOC content showed significantly influence on the sediment AOA community composition (*P* < 0.05), and TC can significance affected the sediment AOB distribution (*P* < 0.05). However, there were not notably correlations between AOMs community structures and environment factors in the water column (Fig. S4c and d).

## Discussion

Many studies have reported the abundance and distribution of ammonia-oxidizing microorganisms in estuarine ecosystems^22,23,29^. However, little was known about the different distribution patterns of AOMs between sediment and water habitats in estuarine environments^33^. In this study, the abundance, diversity and community composition of AOA and AOB in both sediment and water habitats were evaluated to provide insights into the AOMs driving nitrification process in the estuarine environments.

Many previous studies have reported that AOA were more abundant than AOB in both terrestrial and marine systems^16,17,34,35^. However, there were still some contrary results with higher AOB abundance compared to AOA in the sediments of the Colne estuary^29^, aquifer-aquitard system^36^, nitrogen-rich wetlands^37^ and Chongming eastern tidal flat^3^. In the present study, our quantitative PCR results revealed that the archaeal *amoA* gene performed significant higher copy numbers than that of AOB in both sediment and water habitats (*P* < 0.01). The higher AOA abundance in various ecosystems indicate that these organisms are adapted to a broad range of growth conditions, and therefore might have more versatile metabolisms than AOB^34^. It was reported that different affinities for ammonia may cause the differential growth of AOA and AOB. AOA grows at wide ammonia concentrations (0 to 200 μg g^−1^), whereas AOB were prominent only at high concentration (200 μg g^−1^)^24^. Similar phenomena were also found in other habitats, such as dominant AOA detected in ocean water where the ammonia concentration is relatively low^38^, and AOB were more abundant than AOA at high nitrogen and carbon conditions in grasslands^39^. As the ammonia concentrations were relative low in our study (<30 μg g^−1^ or 3 μg ml^−1^, Table S1), it is reasonable that higher AOA abundance was detected in the Yellow River Estuary. Furthermore, previous research had reported that salinity was also a key factor causing the higher abundance of AOB than AOA in estuarine habitats^23^. However, this conclusion was later challenged by a report that the AOA abundance was greater than that of AOB along an estuarine salinity gradient^22^. In the present estuarine ecosystem, there was a wide range of salinity gradients (from 0.1‰ to 27.5‰), which indicated AOA community should have a wider adaptive range of salinity. However, the high abundance of a functional gene does not mean that it is expressed^16^, RNA, protein and even metabolite levels’ experiments are more suitable to evaluate the relative contribution to ammonia oxidization. In addition, the primer selection for the *amoA* gene was also critical for the results of PCR-based methods^40^. Therefore, RNA- or protein-based molecule experiment and ammonia oxidation rates can be used in further study.

An interesting finding in this study is that the AOM abundance showed different distribution pattern between sediment and water habitats. In sediment, AOA and AOB showed consistent distribution pattern (Spearman correlation analysis, *P* < 0.001), with significantly higher abundance in sites SA, SC and SD than sites SB and SE. While in water, AOA and AOB *amoA* gene abundance showed distinct distribution trends (Spearman correlation analysis, *P* > 0.05). Previous study indicated that the microbial community of flowing systems, such as rivers and estuaries, can be influenced by many factors, including environmental factors and flowing disturbance^41^. Here, the different AOM abundance distribution between sediment and water in the Yellow River Estuary was also detected to be affected by environmental factors. In sediment, spearman statistical analyses demonstrated that both total carbon (TC) and ammonium concentrations were significantly and positively correlated with the AOA and AOB abundance (P < 0.05), and TP concentration was significantly and negatively correlated with the AOA and AOB abundance (P < 0.05). The detected correlations were not surprised since ammonium, as the substrate for ammonia oxidizers, can directly influence the distribution of AOA and AOB^24,42^. The results also consisted with previous studies which ammonium can affect the abundance of ammonia-oxidizers in sediment ecosystems^20,43^. Moreover, the relationship between AOM and TC was reasonable. Previous studies reported that AOA can autotrophic and heterotrophic growth by using both inorganic and organic carbon^44,45^. In addition, although Kowalchuk *et al*. reported that AOB belongs to obligate chemolithotrophic bacteria^46^, other study has revealed that AOB could be mixotrophic growth^47^. Zheng *et al*. also found the positive relationship between AOB and carbon content in Chongming eastern intertidal sediments^3^. Therefore, the effect of carbon and nitrogen on AOA and AOB drove a similar abundance distribution pattern in the sediment habitat. To our knowledge, the relationship between AOM abundance and phosphorus has not been studied in sediment. While previous study had reported that higher available phosphorus was adverse to AOA in forest soils^48^. For the water habitat, spearman correlation analyses showed that only AOA abundance had negatively correlation with carbon contents (TC and TOC), which suggested the weak influence of environmental factors on AOMs’ abundance than sediment. Doherty *et al*. found that the microbial community composition of flowing systems like rivers, estuaries, and river plumes is influenced by a broader range of environmental factors (e.g. DOM and POM concentrations), and is more heavily influenced by dispersal and mixing of these microbial communities^41^. Based on their results, we deduced that the abundance of AOM might be also heavily influenced by dispersal and mixing in water, which resulted in a different distribution trends between AOA and AOB.

In the present study, it is indicated that the AOA and AOB community structures performed different habitat distribution patterns in Yellow River estuary. The AOA community exhibited significant difference between sediment and water (R = 0.61, *P* = 0.009, ANOSIM test), while the AOB community showed no significant difference (R = 0.22, *P* = 0.112, ANOSIM test). Combining previous and our own studies, we deduced that there are two potential explanations, including adaptability of AOM and different living ways of AOM. First, the ecological adaptability of AOM is an option to explain their distribution patterns. The AOA community composition of sediment is clearly distinct from that of the overlying water. Our phylogenetic analysis indicated that the Group 1.1b (*Nitrososphaera* cluster) was the most dominant (54.9% of all AOA sequences) species in the estuarine sediment, especially in site SA, SC and SD (63.29%-90.48%). Combining the results from previous studies, *Nitrososphaera* cluster was commonly considered as the dominated species in soils^32,49^. As estuary might mix population of both soil and sediment^50^, therefore, these sequences might be from the upstream terrestrial environments taking by the Yellow River. While in water samples, the Group 1.1a (*Nitrosopumilus* cluster and *Nitrosotalea* cluster) of AOA occupied more percentage, which counted 77.19% of the whole AOA sequences. The dominant distribution of *Nitrosopumilus* cluster was consistent with other estuarine and marine environments, such as San Francisco Bay^23^, the Pearl River estuary^40^ and Hangzhou Bay^51^. While for AOB, *Nitrosospira* cluster occupied higher percentages in both sediment and water samples. Previous study has also reported that the *Nitrosospira* cluster may be more adaptable^52^. The evidences suggested that the AOA species possibly performed more habitat bias than AOB in Yellow River estuary. Secondly, different living ways of AOA and AOB might also influence the community distribution between the sediment and water. Previous study concluded that the composition of particle-associated microbe is rather stable, whereas the free-living community changes rapidly and unregularly^53^. Ma *et al*. speculated that AOA might distribute largely in the free-living state whereas AOB mainly distributed at the particle-attached state^54^. While in our study, we deduced a different opinion which AOA (at least the dominant cluster) might be particle-attached and AOB (at least the predominant genus) was free-living. As site E and B located in the intersection between Yellow River and Bohai Sea, Yellow River water sample (WE) carried larger amounts of soil which was a key origin of sediments of SE and SB. The AOA community showed greater similarity between SE, SB and WE, indicating that AOA might be particle-attached on soil particles of Yellow River water. While AOB community might exchange and connect between overlying water and surface sediment as a free-living state, that makes AOB community showed no significant difference between the two habitats. More powerful evidences should be provided to illuminate the living ways of AOA and AOB in future studies.

The 16S rRNA gene high-throughput sequencing can also reveal the prokaryotic community composition related to ammonia oxidizers (Table S5). Based on the result from our previous study^31^, the archaeal genera related to ammonia oxidizers were *Nitrosopumilus*, *Nitrososphaera, Nitrosoarchaeum* and unclassified *Thaumarchaeota*. The genus *Nitrosopumilus* was the most dominant specie, which accounted for 29.09% of the whole archaeal sequences, and 88.25% of the ammonia oxidizers related sequences. This was generally consistent with our *amoA* gene-based result. For 16S-based bacterial community composition, genera *Nitrosomonas, Nitrosospira, Nitrosococcus* and unclassified *Nitrosomonadaceae* were related to ammonia oxidizers. The unclassified *Nitrosomonadaceae* was the most dominant AOB, which counted 1.66% of the whole bacterial sequences, and 91.09% of the ammonia oxidizers related sequences. *Nitrosomonadaceae* include two genera, *Nitrosomonas* and *Nitrosospira*, but the 16S-based taxonomic result could not further classify them on genus level. Our *amoA*-based results indicated that the most dominant AOB was *Nitrosospira*, followed by *Nitrosomonas*, which had a very clear classification on genus level. Above all, both 16S and *amoA*-based primer sets obtained similar results. However, compared 16S rRNA gene, the *amoA* functional gene had more accurate taxonomic classification result and more direct evidence to identification the ammonia-oxidizing microorganisms. Actually, the differences between 16S rRNA and *amoA* gene have resulted in concerns about accuracy, reproducibility, and contamination in previous studies. Junier *et al*. has summarized all the published primer sets for AOMs’ *amoA* gene amplification^55^, and it has indicated that use of specific 16S rRNA gene primers for studying AOM is not a promising approach. The main pitfall of the 16S rRNA gene as a molecular marker is that it is not necessarily related to the physiology of the target organisms. In addition, Meinhardt *et al*. also insisted that the common used *amoA* gene was more accurate and specific to detect AOMs in environmental samples^56^. Moreover, due to the *amoA*-based clone library method with relative low coverage, some rare ammonia-oxidizing species may be undetected. With the development of the third generation sequencing, the long *amoA* genes may be high-throughput sequenced in future study.

Environmental factors have been widely confirmed to affect microbial distributions in estuarine ecosystems^14,22,26,31^. In the present study, both the Mantel test and RDA indicated carbon contents appeared to be the key explanatory variables influencing the sediment AOM community structures. The relationship between AOM and carbon content (TC and TOC) was reasonable because both AOA and AOB can mixotrophic growth by using inorganic and organic carbon^44,45,47^. The carbon contents showed a significant correlation with both AOA and AOB community composition were also found in intertidal sediments of the Yangtze Estuary^57^, Chinese paddy soils^58^ and reservoir riparian soil^12^. However, there were very few notably correlations between AOMs taxa and environment factors in the water samples. The different responses of AOMs to environmental factors might be explained by the distinct properties of the two habitats. In the vertical direction, although the overlying water and surface sediment are connected, particles and nutrients exchange and the movement of water are reduced by a thin sediment-water interface (SWI)^59,60^. Our previous study also indicated that the SWI might produce a physical barrier between the sediment and overlying water in Yellow River estuary^31^. The SWI makes the sediment and water column to be relative isolated habitats. Consequently, sediment habitat characterized more nutrients and less environmental fluctuations compared to the water column. The flowing disturbance from Yellow River led to drastic fluctuation of water column as well as its microbes and environmental variables. Thus, randomicity from the flowing disturbance might be more powerful than the environmental selection in water column of Yellow River estuary. In addition to carbon, other factors such as pH^12,14^, temperature^61^, total phosphorus^13^ and NO_3_^−^N^52^ might also influence the community composition of AOA and AOB. However, no other measured factors showed a significant correlation with the AOM composition in the Yellow River Estuary, suggesting that carbon might be critical factors in shaping the ammonia-oxidizers communities in this estuary.

Taken together, we confirmed that there were obvious distinct abundance, community structure, and distribution patterns between surface sediment and overlying water column in Yellow River estuary. Our results indicated that habitats heterogeneity has significant influence on AOMs community distributions, even in the closely related sediment and overlaying water habitats of the estuarine ecosystem. Habitat features, environmental selections and physiological characteristics codetermined the distribution patterns of AOMs. Nevertheless, single season’s sampling and DNA-based study cannot completely reflect the ammonia oxidizing microbial ecology in Yellow River estuary, and we expect to perform some time-serial, large-scale sampling and RNA-based researches around this area in the future.

## Material and Methods

### Samples collection and physicochemical analysis

The Yellow River estuary lies between Laizhou Bay and Bohai Bay, and it is one of the three major estuaries in China. The details of sampling and physicochemical characteristics have been described in our previous study^31^. Briefly, surface sediment samples (approximately 0–5 cm) and the overlying water samples from five different sites (A to E) were collected around the Yellow River estuary. After being evenly mixed of three replicates, the sediment cores and water samples were transported on ice to the laboratory, immediately. The physicochemical factors, including depth (Dep), pH, salinity (Sal), dissolved oxygen (DO), total carbon (TC), total nitrogen (TN), total phosphorus (TP), total organic carbon (TOC), nitrate (NO_3_^−^) andammonia (NH_4_^+^) concentrations, were shown in Table S1.

### DNA extraction and PCR amplification

The total genomic DNA of each water or sediment sample was extracted using E.N.Z.A.^TM^ Water DNA Kit and Soil DNA Kit (Omega, USA) according to the manufacturer’s instructions. The quality of the extracted DNA was examined by 1.0% (*w/v*) agarose gel electrophoresis. Triplicate DNA extracts of each sample were pooled together and stored at −20 °C for further analysis. The *amoA* genes fragments were amplified using the primer pairs of Arch-*amoA*F/R for targeting AOA^33^ and *amoA*-1F/2 R for targeting AOB^4^. The primer sequences and the PCR conditions were listed in Table S2.

### Cloning and sequencing

The appropriately sized fragments were separated by electrophoresis in 1% agarose gels and then purified with Agarose Gel DNA Retrieved Kit (Solarbio, China). The purified fragments were ligated to pMD18-T vectors (Takara, Japan) and then transformed into *E. coli* DH5α competent cell (Tiangen, China) in accordance with the manufacturer’s instructions. Colonies were cultured on Lysogeny broth (LB) agar plates containing ampicillin (100 μg/ml), more than 50 positive clones were randomly selected from each library and then were sequenced using ABI PRISM 3730 automated sequencer (Applied Biosystems, USA).

### Quantitative PCR

The primers described above were used to quantify the copy numbers of archaeal and bacterial *amoA* genes (Table S2). Amplification reactions were carried out with the SYBR Premix Ex *Taq* (Takara, Japan) in a total volume of 20 µl, and the reaction composition and cycling conditions were in accordance with the manual. Standard curves were obtained using serial dilutions of a known copy number of plasmids containing the *amoA* gene fragment, these are linearized and their abundance ranged from 1.54 × 10^2^ to 1.54 × 10^8^ copies/μl for bacterial *amoA* genes (R^2^ = 0.998, E = 94.9%), and from 2.24 × 10^2^ to 2.24 × 10^8^ copies/μl for archaeal *amoA* genes (R^2^ = 0.992, E = 105.2%), respectively. All samples were analyzed in triplicate. In all experiments, negative controls were subjected to the same qPCR procedure to exclude any possible contaminations.

### Data analyses and statistical tests

For AOA and AOB, clones with more than 85% sequence similarity were grouped into a same operational taxonomic units (OTU) by Mothur software^62^, and the most abundant representative sequence of each OTU was used for phylogenetic analysis. Then the phylogenetic trees were constructed with the neighbor-joining method with 1000 bootstrap repetitions to estimate the confidence of the tree topologies using MEGA, version 5.1^63^. The α-diversity estimators were performed to assess the richness and evenness of taxa contained within an individual community, which included diversity indexes (Shannon-Wiener index), richness estimator (Chao1) and coverage rate. The β-diversity was assessed to reveal the community composition or structure similarity of different samples, included Principal coordinate analysis (PCoA), analysis of similarities and cluster analysis (CA). Principal coordinate analysis (PCoA) and analysis of similarities (ANOSIM) were used to measure community similarity based on the algorithm of the Bray-Curtis matrix, and 999 Monte Carlo permutations were used to assess the statistical significance of diversity metrics. Visualization of β-diversity was performed by cluster analysis (CA). Both α-diversity and β-diversity were calculated using PAleontological STatistics (PAST) software.

Redundancy analysis (RDA) between the environmental factors and microbial communities was performed using CANOCO 5 software (Microcomputer Power, USA) based on the result of detrended correspondence analysis (DCA). Environmental factors were forward selected for significance tests using 999 Monte Carlo permutations. Mantel tests of the environmental factors and cluster-based distance matrixes were also performed in PAST software with 9999 Monte Carlo permutations for the significance tests. Spearman correlation analyses and significance tests were determined using SPSS statistics software (version 19, IBM, USA).

### Nucleotide sequence accession numbers

The sequences of *amoA* gene fragments reported in this study have been deposited in GenBank under the accession numbers KP781273-KP781558 for sediment AOA, KP781023-KP781272 and KX279988-KX280011 for sediment AOB, KY130001-KY130171 for water AOA and KY130172-KY130403 for water AOB.

## Electronic supplementary material

Supplementary materials
