# Supplementary material for: Distinct distribution patterns of ammonia-oxidizing archaea and bacteria in sediment and water column of the Yellow River estuary
Source: Sci Rep. 2018 Jan 25;8:1584. doi: 10.1038/s41598-018-20044-6 (PMC5785527; doi:10.1038/s41598-018-20044-6)
Supplement: Supplementary file 1 — Supplementary materials [file 41598_2018_20044_MOESM1_ESM.doc]

**Supplementary materials**

**Distinct distribution patterns of ammonia-oxidizing archaea and bacteria in sediment and water column of the Yellow River estuary**

Mingcong Li^1,2^, Guangshan Wei^1,4,5^, Wenchong Shi^1,2^, Zhongtao Sun^1^, Han Li^1,2^, Xiaoyun Wang^1,2^, Zheng Gao^1,2,3^

^1^State Key Laboratory of Crop Biology, Shandong Agricultural University, Tai'an 271018, China. ^2^College of Life Sciences, Shandong Agricultural University, Tai'an 271018, China. ^3^Department of Botany and Microbiology, Institute for Environmental Genomics, University of Oklahoma, Norman, OK, USA. ^4^Key Laboratory of Marine Genetic Resources, Third Institute of Oceanography, SOA, Xiamen 361005, China. ^5^South China Sea Resource Exploitation and Protection Collaborative Innovation Center (SCS-REPIC), Sun Yat-Sen University, Guangzhou 510275, China. M. L. and G. W. contributed equally to this work. Correspondence and requests for materials should be addressed to Z. G. **(**gaozheng@sdau.edu.cn) or X. W. (xyunwang@sdau.edu.cn)


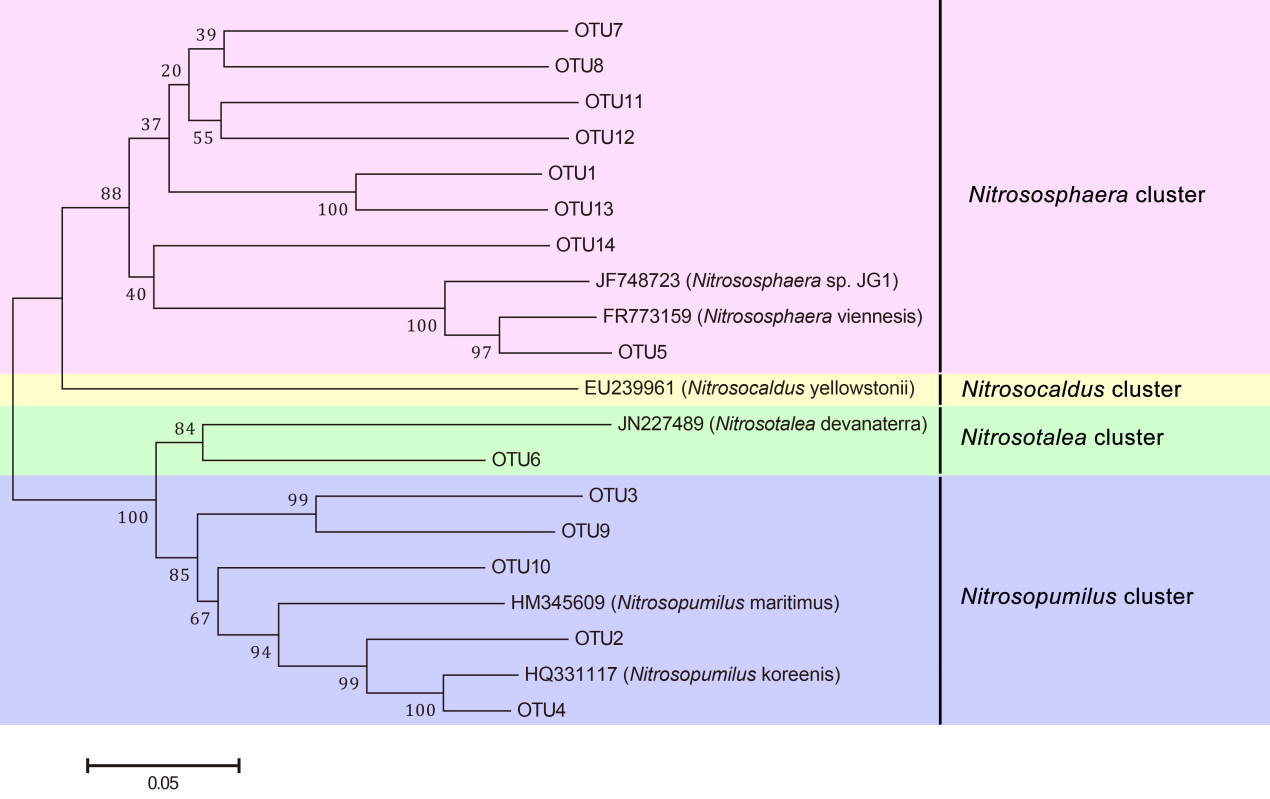


**Fig. S1.** **Neighbor-joining phylogenetic tree of AOA *amoA* gene sequences of representative OTUs and known AOA isolated strains.** Lineage names are derived from the genus name of the first cultured representative of each respective group. Bootstrap values greater than 50% of 1,000 replicates are shown, and the scale bar represents 5% sequence divergence. GenBank accession numbers are shown for sequences from other studies.


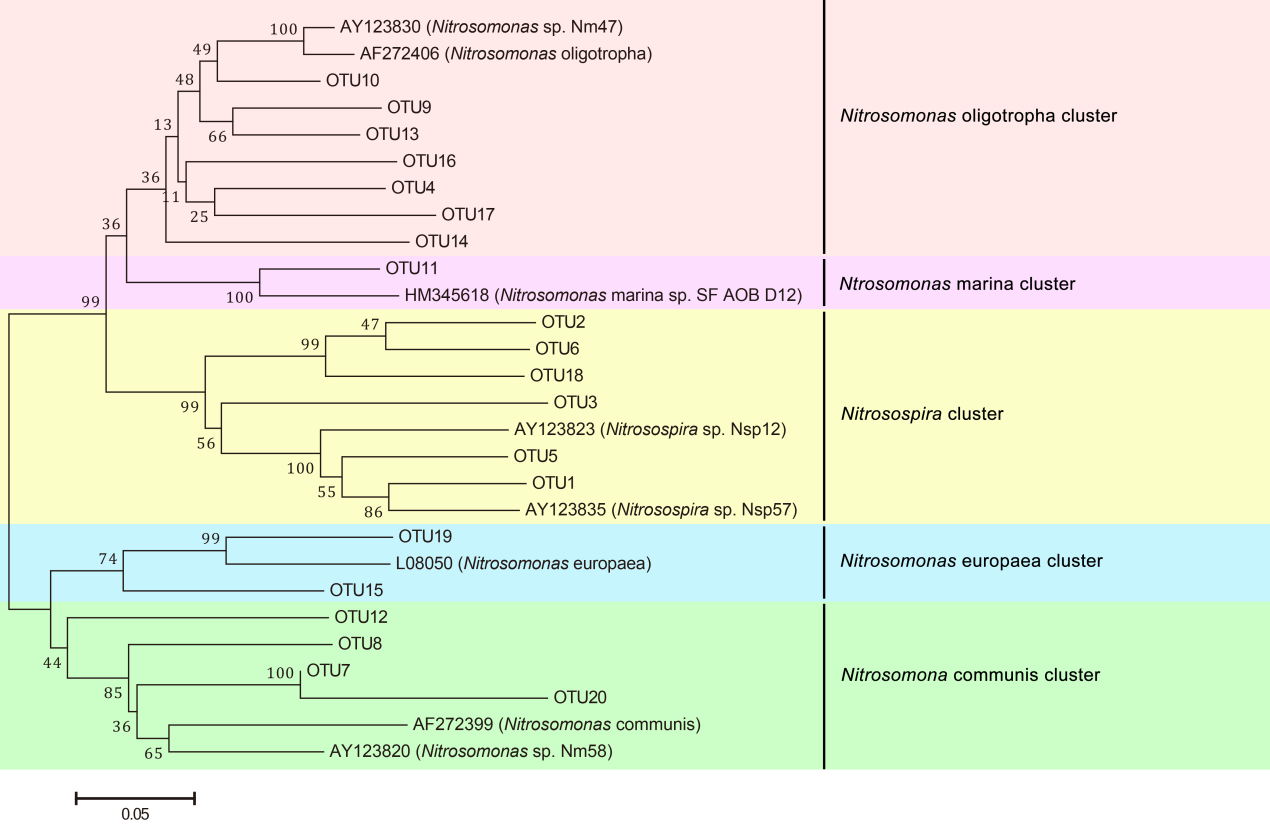


**Fig. S2.** **Neighbor-joining phylogenetic tree of AOB *amoA* gene sequences of representative OTUs and known AOB isolated strains.** Lineage names are derived from the genus name of the first cultured representative of each respective group. Bootstrap values greater than 50% of 1,000 replicates are shown, and the scale bar represents 5% sequence divergence. GenBank accession numbers are shown for sequences from other studies.

**
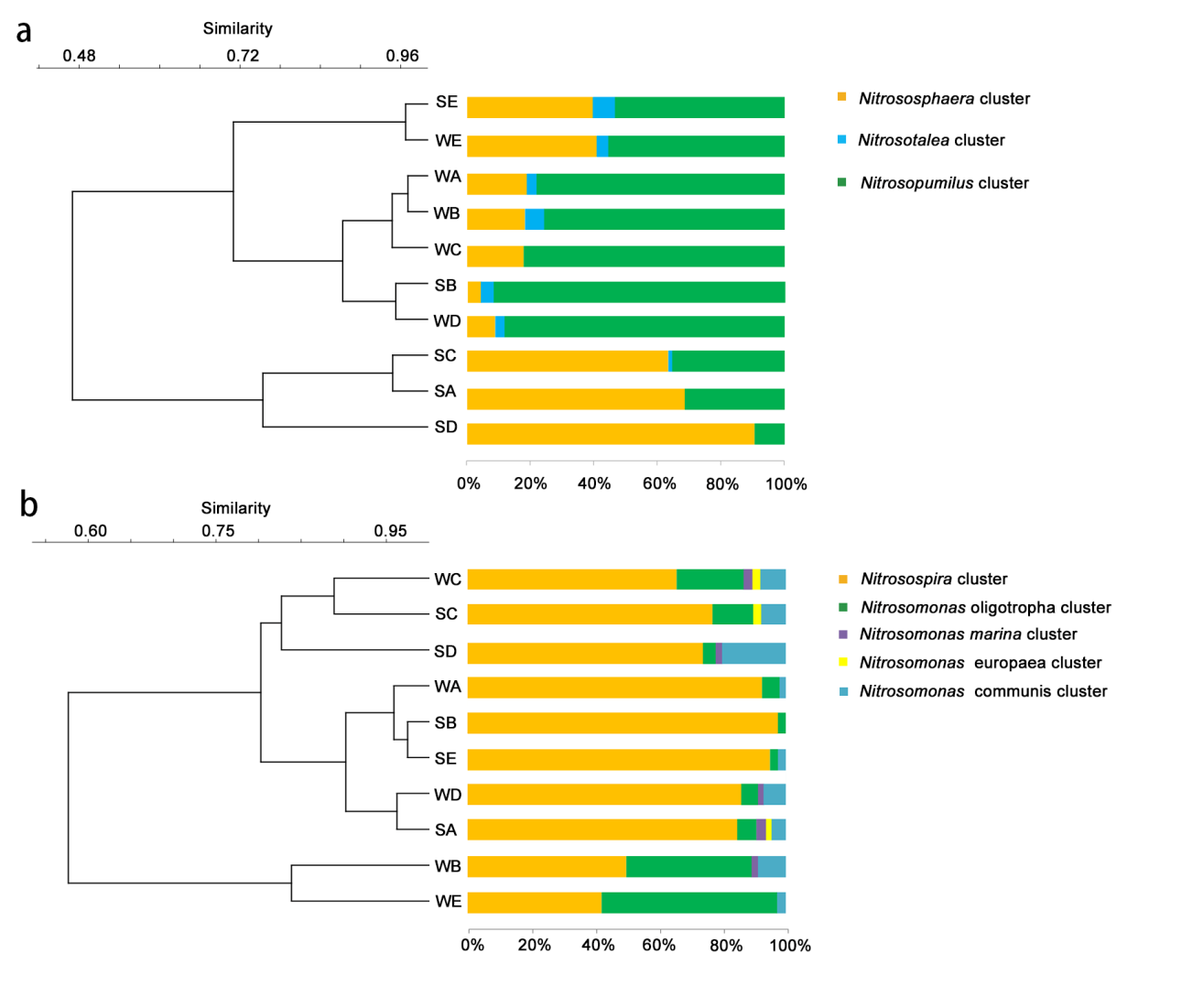
**

**Fig. S3. Community compositions of (a) AOA and (b) AOB at cluster level in sediment and water column.** Hierarchical clustering of samples are based on the Bray-Curtis similarity algorithm.


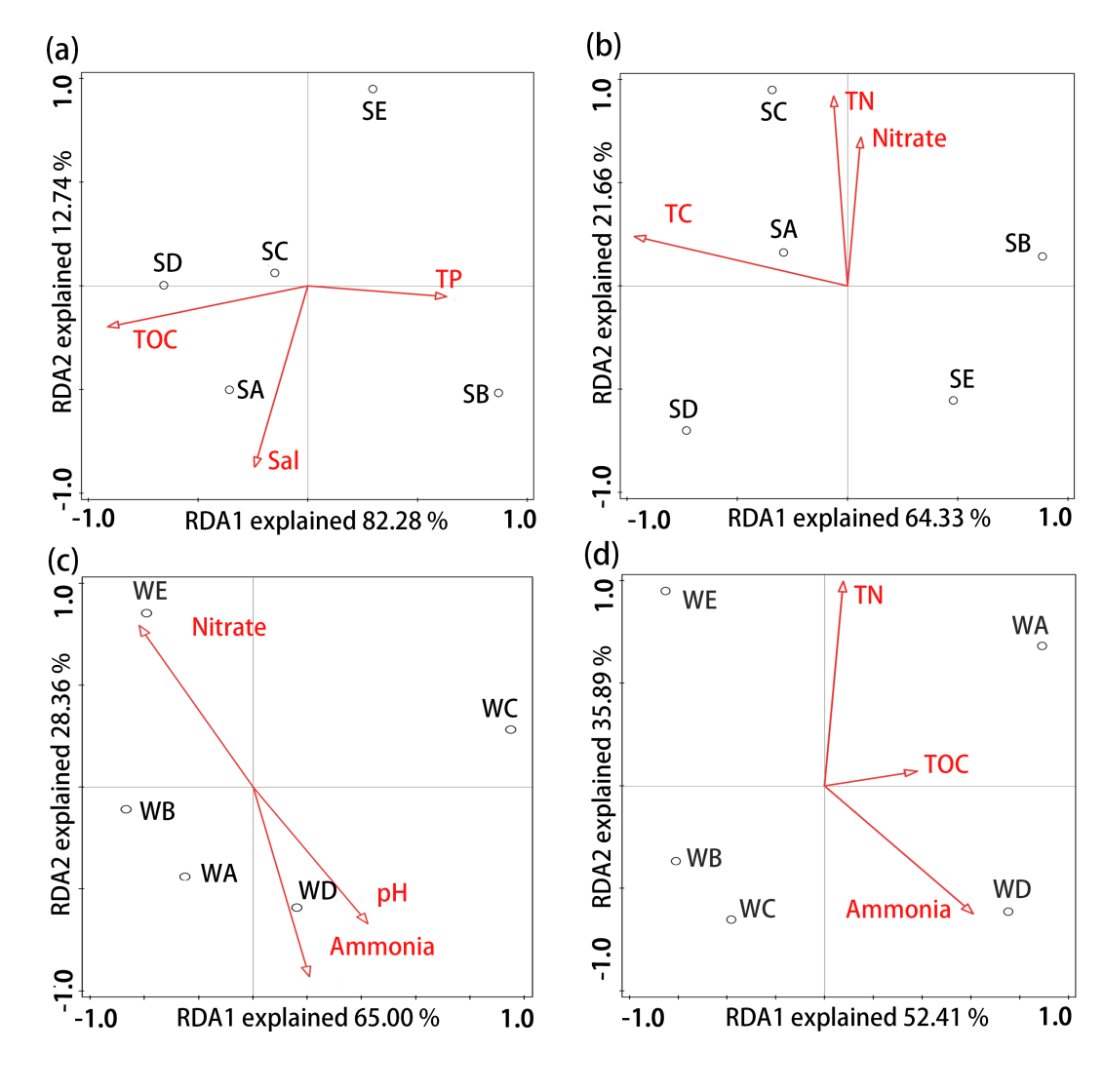


**Fig.** **S4. RDA ordination plots of AOMs communities and representative environmental factors.** RDA analyses are calculated based on cluster level data and environmental variables. Circles in the plots show the sampling sites, and arrows indicate environmental factors. Correlations between environmental factors and RDA axes are represented by the length and angle of arrows.

**Table S1** Physicochemical characteristics of the water and sediment samples

| **Type** | **Sites** | **Dep** | **pH** | **Sal** | **DO** | **TC** | **TN** | **TP** | **TOC** | **NO_3_^-^** | **NH_4_^+^** |
| --- | --- | --- | --- | --- | --- | --- | --- | --- | --- | --- | --- |
| **Sediment** | **A** | 7.5 | 8.0 | 25.5 | 7.88 | 21.9 | 1.36 | 164.61 | 4.24 | 0.900 | 19.46 |
|  | **B** | 0.9 | 8.1 | 26.8 | 6.25 | 10.2 | 1.14 | 171.82 | 1.31 | 0.601 | 7.08 |
|  | **C** | 2.4 | 7.8 | 27.5 | 6.01 | 26.0 | 1.39 | 100.22 | 5.17 | 0.661 | 27.73 |
|  | **D** | 2.7 | 7.9 | 25.7 | 8.52 | 25.6 | 0.82 | 110.75 | 6.05 | 0.233 | 21.47 |
|  | **E** | 0.9 | 8.4 | 0.1 | 6.53 | 12.8 | 0.87 | 168.48 | 1.51 | 0.352 | 12.58 |
| **Water** | **A** | 0.5 | 8.0 | 27.3 | 7.93 | 61.9 | 8.64 | 0.965 | 22.30 | 0.200 | 1.98 |
|  | **B** | 0.5 | 8.1 | 25.2 | 7.06 | 53.4 | 6.51 | 0.037 | 20.15 | 0.107 | 1.93 |
|  | **C** | 0.5 | 8.1 | 26.8 | 7.21 | 35.6 | 5.90 | 0.168 | 11.80 | 0.057 | 1.71 |
|  | **D** | 0.5 | 8.0 | 24.7 | 7.15 | 40.4 | 6.10 | 0.001 | 12.61 | 0.085 | 2.06 |
|  | **E** | 0.5 | 7.6 | 0.1 | 9.06 | 24.8 | 8.80 | 0.029 | 9.29 | 1.706 | 0.76 |

Dep:depth (m); Sal: salinity (ppt), here is the salinity of bottom water for sediments; DO: dissolved oxygen (mg/L), here is the DO of bottom water for sediments; TC: total carbon (mg/L for water, g/kg for sediment); TN: total nitrogen (mg/L for water, g/kg for sediment); TP: total phosphorus (mg/L for water, mg/kg for sediment); TOC: total organic carbon (mg/L for water, g/kg for sediment); NO_3_^-^: nitrate nitrogen (mg/L for water, mg/kg for wet sediment); NH_4_^+^: ammonium nitrogen (mg/L for water, mg/kg for wet sediment)

**Table S2** Primers and PCR conditions for amplification of *amoA*

| Target gene | Primer | Sequence(5’-3’) | Length of product(bp) | PCR program | Reference |
| --- | --- | --- | --- | --- | --- |
| *amoA*  AOA | Arch-*amoA*F | STAATGGTCTGGCTTAGACG | 635 | 94 ℃, 5 min (94 ℃, 40 s ; 53 ℃, 40 s; 72 ℃, 50 s) × 32; 72 ℃, 10 min | Francis et al. 2005 |
|  | Arch-*amoA*R | GCGGCCATCCATCTGTATGT |  |  |  |
| *amoA*  AOB | *amoA*-1F | GGGGTTTCTACTGGTGGT | 491 | 94 ℃, 5 min (94 ℃, 40 s ; 56 ℃, 40 s; 72 ℃, 50 s) × 32; 72 ℃, 10 min | Rotthauwe et al. 1997 |
|  | *amoA*-2R | CCCCTCKGSAAAGCCTTCTTC |  |  |  |

Abbreviations: AOA, ammonia-oxidizing archaea; AOB, ammonia-oxidizing bacteria; PCR, polymerase chain reaction

**Table S3** Mantel tests between sediment AOMs' community (cluster level) and environmental variables in Yellow River estuary.

| AOA community | | | AOB community | | |
| --- | --- | --- | --- | --- | --- |
| Variable | R | *P* | Variable | R | *P* |
| pH | -0.1242 | 0.5317 | pH | 0.2047 | 0.2747 |
| Sal | -0.2290 | 0.5660 | Sal | -0.2144 | 0.7700 |
| DO | 0.1619 | 0.3174 | DO | 0.2439 | 0.2350 |
| **TC** | **0.6667** | **0.0371** | **TC** | **0.7203** | **0.0473** |
| TN | -0.2002 | 0.8023 | TN | -0.0433 | 0.3895 |
| TP | 0.0431 | 0.4264 | TP | 0.6848 | 0.0544 |
| **TOC** | **0.6427** | **0.0166** | **TOC** | **0.8731** | **0.0076** |
| Nitrate | -0.3038 | 0.7819 | Nitrate | -0.1942 | 0.6746 |
| Ammonium | 0.5420 | 0.0690 | Ammonium | 0.5355 | 0.0828 |

**Table S4** Mantel tests between water AOMs' community (cluster level) and environmental variables in Yellow River estuary.

| AOA community | | | AOB community | | |
| --- | --- | --- | --- | --- | --- |
| Variable | R | *P* | Variable | R | *P* |
| pH | 0.8641 | 0.1943 | pH | 0.3896 | 0.0987 |
| Sal | 0.9160 | 0.1596 | Sal | 0.4233 | 0.0764 |
| DO | 0.7910 | 0.1516 | DO | 0.2434 | 0.2739 |
| TC | 0.2360 | 0.3969 | TC | 0.0604 | 0.3440 |
| TN | 0.2385 | 0.1051 | TN | -0.1519 | 0.5346 |
| TP | -0.4067 | 0.9579 | TP | 0.0616 | 0.2861 |
| TOC | -0.0089 | 0.5079 | TOC | -0.1456 | 0.5931 |
| Nitrate | 0.9253 | 0.0784 | Nitrate | 0.4080 | 0.1699 |
| Ammonium | 0.9157 | 0.0502 | Ammonium | 0.4380 | 0.0665 |

**Table S5 The 16S rDNA high-throughput sequencing based sequence number of AOB and AOA among sampling sites between sediment and water column.**

| **Potential AOMs** | | **Sequence number of AOMs among different sampling sites** | | | | | | | | | |
| --- | --- | --- | --- | --- | --- | --- | --- | --- | --- | --- | --- |
| **AOB** | **SA** | | **SB** | **SC** | **SD** | **SE** | **WA** | **WB** | **WC** | **WD** | **WE** |
| *Nitrosococcus* | 54 | | 11 | 26 | 20 | 18 | 4 | 5 | 2 | 8 | 3 |
| *Nitrosomonas* | 6 | | 40 | 24 | 29 | 51 | 1 | 2 | 4 | 5 | 10 |
| *Nitrosospira* | 0 | | 0 | 0 | 0 | 2 | 0 | 0 | 0 | 2 | 0 |
| Unclassified  *Nitrosomonadaceae* | 1 | | 47 | 20 | 4 | 40 | 1 | 1 | 2 | 2 | 5 |
| Total seqs | 16949 | | 23521 | 20588 | 18076 | 19844 | 19170 | 20415 | 17048 | 29361 | 16864 |
| **AOA** | **SA** | | **SB** | **SC** | **SD** | **SE** | **WA** | **WB** | **WC** | **WD** | **WE** |
| *Nitrosoarchaeum* | 13 | | 664 | 648 | 33 | 789 | 13 | 96 | 19 | 21 | 136 |
| *Nitrosopumilus* | 3607 | | 50 | 1392 | 376 | 13 | 4584 | 5982 | 5030 | 4223 | 5 |
| *Nitrososphaera* | 15 | | 47 | 142 | 42 | 85 | 17 | 6 | 7 | 5 | 308 |
| Unclassified *Thaumarchaeota* | 48 | | 23 | 40 | 29 | 41 | 12 | 7 | 9 | 9 | 38 |
| Total seqs | 7895 | | 7252 | 8556 | 6939 | 8159 | 9011 | 12525 | 9969 | 9278 | 7265 |

**Reference**

Francis, C. A.; Roberts, K. J.; Beman, J. M.; Santoro, A. E.; Oakley, B. B., Ubiquity and diversity of ammonia-oxidizing archaea in water columns and sediments of the ocean. *Proceedings of the National Academy of Sciences of the United States of America* **2005,** 102, (41), 14683-14688.

Rotthauwe, J.-H.; Witzel, K.-P.; Liesack, W., The ammonia monooxygenase structural gene amoA as a functional marker: molecular fine-scale analysis of natural ammonia-oxidizing populations. *Applied and Environmental Microbiology* **1997,** 63, (12), 4704-4712.
